# Changes in Food Security and Diet Quality After the 2021 SNAP Benefit Increase in Massachusetts, USA

**DOI:** 10.3390/nu18111729

**Published:** 2026-05-28

**Authors:** Mary Kathryn Poole, Lauren G. Fiechtner, Erin O’Dwyer, Cara F. Ruggiero, Eric B. Rimm, Matthew M. Lee, Kate Adams, Erica L. Kenney

**Affiliations:** 1 Division of Gastroenterology, Hepatology, and Nutrition, Department of Pediatrics, Stanford University School of Medicine, Stanford, CA 94305, USA; 2 Department of Nutrition, Harvard T.H. Chan School of Public Health, Boston, MA 02115, USA; 3 Division of General Academic Pediatrics, Department of Pediatrics, Mass General for Children, Boston, MA 02114, USA; 4 Division of Gastroenterology and Nutrition, Department of Pediatrics, Mass General for Children, Boston, MA 02114, USA; 5 The Greater Boston Food Bank, Boston, MA 02118, USA; 6 Department of Epidemiology, Harvard T.H. Chan School of Public Health, Boston, MA 02115, USA; 7 Department of Social and Behavioral Sciences, Harvard T.H. Chan School of Public Health, Boston, MA 02115, USA

**Keywords:** Supplemental Nutrition Assistance Program, food stamps, food security, diet quality, Thrifty Food Plan

## Abstract

Background/Objectives: The Supplemental Nutrition Assistance Program (SNAP) is the largest food assistance program in the United States providing income-eligible households with cash-like assistance to spend on food. In October 2021, a historic policy change permanently increased benefit amounts by 21 percent. This study assessed differences in diet quality and food security, supplemented by participant descriptions of using SNAP, among adults with low incomes residing in the state of Massachusetts from before (October 2020–January 2021) to after (December 2021–February 2022) the benefit increases. Methods: Derived from The Greater Boston Food Bank’s Annual Statewide Survey, our sample included adults with household incomes ≤300% of the federal poverty level who completed diet and food security measures. We calculated Prime Diet Quality Scores (PDQSs), with higher scores reflecting more nutritious diets on a scale of 0–70. Adjusted difference-in-difference regression models evaluated differences in PDQS and food security between SNAP participants and non-participants from before to after the benefit increases. Reflexive thematic analysis of write-in responses summarized experiences with SNAP. Results: Complete data were available for 1051 respondents before and 801 respondents after SNAP benefit increases. We found no significant differences in diet quality or household food security for SNAP participants, compared to non-participants, from before to after the benefit increases. In write-in comments, respondents expressed gratitude for increased benefits but also fear of them being rescinded. Increased benefits helped some better meet food needs, yet many noted rising costs of living prevented benefits from stretching as far. Conclusions: Increased SNAP benefits did not impact food security or diet quality among this sample. SNAP benefits may need to be further increased to meet the nutritional needs of families.

## 1. Introduction

Food insecurity—where a household experiences insufficiency or uncertainty in obtaining adequate food for its members [1]—is a persistent and substantial public health issue worldwide, affecting an estimated 28% of the global population [2]. In the United States (U.S.), the prevalence of food insecurity has stubbornly remained above 10% for the last several decades, and racial/ethnic disparities have persisted [3]. Food insecurity is associated with higher healthcare costs [4], mental health conditions [5], and chronic disease including cardiovascular disease, type 2 diabetes, and certain cancers [6]. These chronic conditions are potentially mediated through food insecurity’s adverse effects on diet quality [7], a symptom of the structural barriers to accessing affordable, nutritious food.

Worldwide, different countries utilize a range of strategies to address food insecurity. Some focus on income directly, rather than food insecurity in particular, i.e., cash transfer or income support programs; other approaches include delivering foods directly to households in need [8]. To ameliorate hunger and food insecurity in the U.S., the government uses a mix of these approaches. While the U.S. operates several programs that provide meals or direct nutritional assistance to children, the largest program (and the only one that supports households without children) is the Supplemental Nutrition Assistance Program (SNAP) [9], which provides low-income households with cash-like benefits that can only be spent on food and are meant to supplement, but not fully account for, the household’s food budget [10]. Yet, while SNAP substantially reduces the risk of household food insecurity [11,12], SNAP benefits do not fully close the gap between low-income households’ financial resources and the monthly costs of sufficient, nutritious food [13,14]. Nearly half of SNAP participants still report food insecurity [3] and participation in SNAP does not consistently improve diet quality [15].

It is not clear, however, whether increasing the cash value of SNAP benefits would result in improvements in food security and diet quality. SNAP benefit amounts are calculated for each household based on its characteristics (e.g., size, income, disability) and a maximum amount given in the U.S. Department of Agriculture’s Thrifty Food Plan, a food plan developed to meet dietary needs at the lowest possible cost in a U.S. context [16]. Since its development in the 1970s, the Thrifty Food Plan had not been re-evaluated to take into account changes in the food supply and food preparation practices, leading to criticisms that SNAP benefits were not adequate for supporting a nutritious diet [17]. In 2021, however, the Thrifty Food Plan was finally revised, resulting in a 21% increase in benefits, which translated to an increase of approximately $36/month or $1.20/day per person [18]. While increasing food assistance generally reduces food insecurity [19], it is unclear whether this specific increase positively impacted food insecurity and diet quality; only one study has so far evaluated potential impacts of the Thrifty Food Plan revision on food insecurity and diet quality among adults with low incomes [20].

Building on this one prior evaluation of the Thrifty Food Plan revision, this study uses a difference-in-differences approach, supplemented by a reflexive thematic analysis of study participants’ written comments about experience with using SNAP, to evaluate whether adult diet quality and household food security changed from before to after the Thrifty Food Plan-related benefit increase in one U.S. state (Massachusetts).

## 2. Materials and Methods

### 2.1. Study Design and Sample

**Figure 1:**
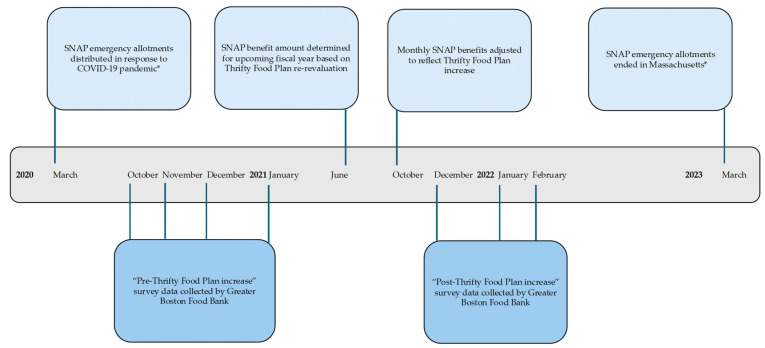
Timeline of data collection relative to changes in SNAP benefits (2020–2023). * SNAP emergency allotments are displayed to demonstrate that SNAP participants in both the pre- and post-Thrifty Food Plan increase samples received these temporary increased benefit amounts separate from the Thrifty Food Plan revision. SNAP = Supplemental Nutrition Assistance Program.

We used repeated cross-sectional data from The Greater Boston Food Bank’s Annual Statewide Survey on Food Insecurity, Equity & Access (The Greater Boston Food Bank, Boston, MA, USA) designed to assess utilization of nutrition assistance programs and prevalence of household food insecurity among a representative sample of adults with low incomes residing in Massachusetts [21]. The Greater Boston Food Bank collected data for the Annual Statewide Survey on Food Insecurity, Equity & Access at two time points: October 2020–January 2021 (pre-Thrifty Food Plan increase) and December 2021–February 2022 (post-Thrifty Food Plan increase). See Figure 1 for the timing of data collection relative to the Thrifty Food Plan policy change and subsequent increases to SNAP benefits. Eligible participants included individuals who were 18 years or older and lived in Massachusetts at the time of data collection. The Greater Boston Food Bank recruited participants, using Qualtrics research panels [22], by oversampling adults with low incomes, to reach a target sample of 3000 adults reflecting the sociodemographic composition of Massachusetts residents. Utilization of a food pantry was not a criterion for either the sampling frame or inclusion in the sample. Further details of sampling methods are available elsewhere [21,23,24,25].

**Figure 2:**
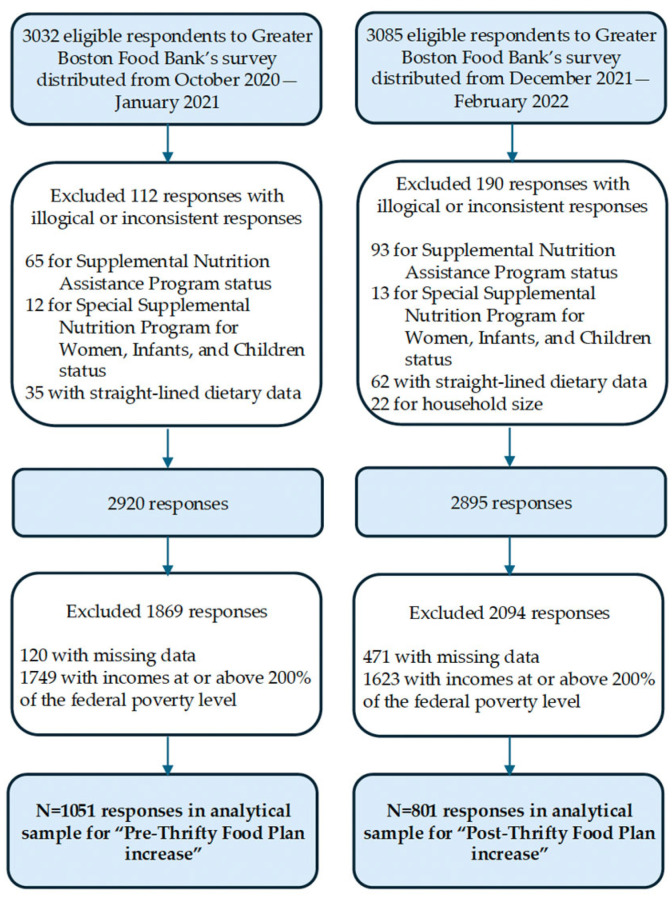
Flow diagram of respondents to Greater Boston Food Bank’s Annual Statewide Survey on Food Insecurity, Equity & Access.

The Greater Boston Food Bank collected survey responses from 3032 respondents before and 3085 respondents after the SNAP benefit increase. We excluded 302 respondents with inconsistent responses to survey questions about participation in food assistance programs including SNAP and/or the Special Supplemental Nutrition Assistance Program for Women, Infants, and Children, use of food pantries, and dietary intake (see Figure 2 for participant flow diagram presented by survey year). We also excluded 591 respondents with missing data on exposures, outcomes, and covariates. Next, to minimize confounding of diet quality and food security differences by income, we restricted our sample to respondents with low incomes, expressed as a percentage of the federal poverty level, by excluding 3372 respondents with incomes above 300% of the federal poverty level. We arrived at an analytical sample of 1051 adults at pre-Thrifty Food Plan increase and 801 at post-Thrifty Food Plan increase for the quantitative analyses. We analyzed a subset of the sample comprising SNAP-enrolled respondents with valid responses to open-ended questions (*n* = 122 at pre-Thrifty Food Plan increase, *n* = 82 at post-Thrifty Food Plan increase) for the qualitative analysis.

### 2.2. Measures

Eligible participants completed an online Qualtrics survey with closed- and open-ended questions about diet, household food security, participation in and experiences with nutrition assistance programs, and demographic information.

#### 2.2.1. Outcomes

To measure diet quality, the Greater Boston Food Bank’s survey incorporated the Rapid Prime Diet Quality Screener [26], a brief measure that captures how frequently respondents consumed 14 types of foods in the last 30 days. Respondents reported how often (less than once per week, once per week, two to four times per week, nearly daily or daily, or twice or more per day) they consumed the following foods: (1) processed meats; (2) beef, pork, or lamb; (3) fish; (4) full-fat dairy products; (5) fast food or take-out, pizza, frozen dinners, restaurant meals; (6) soda, soft drinks, sports or energy drinks; (7) white bread, white rice, white pasta; (8) whole-grain bread, brown rice, whole grain pasta; (9) sweets and desserts; (10) beans, lentils, chickpeas, tofu; (11) vegetables; (12) whole fruits; (13) peanut butter and nuts; and (14) beer, wine, or liquor. We used these frequencies to create a Prime Diet Quality Score (“Overall” PDQS) and sub-scores for intake of more nutritious foods (“Healthy” PDQS) and less nutritious foods (“Unhealthy” PDQS) based on an existing scoring algorithm [27].

To measure household food insecurity, the Greater Boston Food Bank’s survey included the U.S. Department of Agriculture’s 6-item Short-Form Food Security Module [28], a series of questions about access to food and food-related stress, to measure household food security in the last 30 days. Food security status (food secure, food insecure) was estimated by the number of affirmative responses (i.e., often or sometimes true) to survey questions in accordance with the U.S. Department of Agriculture’s scoring guidance.

Experiences with using SNAP were measured by responses to an optional open-ended survey question (“Any other comments about using SNAP?”).

#### 2.2.2. Exposures

Greater Boston Food Bank survey respondents reported whether anyone in their household participated in SNAP in the last 30 days (yes/no).

#### 2.2.3. Covariates

Respondents completed Greater Boston Food Bank survey questions about sociodemographic information for themselves and their household. Individual demographic information included age in years (18–34, 35–54, 55–64, 65 or older), gender (male, female), educational attainment (some high school or less, high school graduate or General Educational Development, some college, Associate’s degree, Bachelor’s degree, graduate degree), race or ethnicity (non-Hispanic White, non-Hispanic Black or African American, Hispanic/Latino or Spanish origin, non-Hispanic Asian, Other), and employment status (currently employed or unemployed). Household demographic information included household size, presence of children in household (yes/no), annual household income (<$10,000, $10,000–24,999, $25,000–49,999, $50,000–99,999, $100,000–149,999, $150,000–199,999, ≥$200,000), region in Massachusetts (Central, Western, Eastern), participation in SNAP in the last 30 days (yes/no), participation in the Special Supplemental Nutrition Program for Women, Infants, and Children in the last 30 days (yes/no), and use of a food pantry in the last 30 days (yes/no). Income and household size were combined to estimate household income as a percentage of the federal poverty level (<100%, ≤100% to <200%, ≥200%). Households with incomes less than 200% of the federal poverty level are eligible for SNAP in Massachusetts.

### 2.3. Analysis

We employed descriptive statistics to summarize means and frequencies of PDQSs, household food insecurity, dietary intake, and sociodemographic information. Using t-tests for continuous variables and chi-square tests for categorical variables, we tested for unadjusted differences by participation in SNAP among respondents before and after the benefit increase following the Thrifty Food Plan reevaluation.

The study team used a difference-in-differences approach to explore differences in diet quality and household food insecurity among SNAP-eligible adults from approximately one year before (October 2020–January 2021) to several months after the Thrifty Food Plan reevaluation and the subsequent SNAP benefit increase (December 2021–February 2022). For the outcome of diet quality scores, we used a linear regression model with an indicator for participation in SNAP (yes/no), an indicator for time (pre/post-Thrifty Food Plan increase), and an interaction term for SNAP and time. For the outcome of household food insecurity, we used a linear probability regression model with an indicator for participation in SNAP (yes/no), an indicator for time (pre/post-Thrifty Food Plan increase), and an interaction term for SNAP and time. Both models were adjusted for all other covariates including age, gender, educational attainment, race or ethnicity, employment status, household food insecurity, household size, presence of children in the household, % of federal poverty level, region, participation in the Special Supplemental Nutrition Program for Women, Infants, and Children, and use of a food pantry. We implemented the quantitative analyses in SAS Version 9.4 [29], applied survey weights to account for the sampling design, and reported robust standard errors.

We deemed the difference-in-differences approach to be an appropriate analytical approach for our research aims despite being unable to formally test for violation of the parallel trends assumption. However, pre-pandemic trends in diet quality [30] and food security [31] did not differ for households with low incomes that used SNAP and those that did not. Moreover, there is no evidence of exposure to an event during 2020–2022 that would have disproportionately impacted SNAP households vs. non-SNAP households with low incomes that could explain differences in diet quality or food security. For example, while many households with low incomes accessed pandemic-era food assistance programs (e.g., food pantries, food boxes, free school meals) [32,33], program impacts were not expected to differ by household participation in SNAP.

As a supplementary analysis, we used a difference-in-differences approach to test for differences in the intake of each food category used to calculate the PDQS from before to after the revised Thrifty Food Plan. Sensitivity analyses explored whether the impact of the Thrifty Food Plan increase differed for households with children, which experienced substantial food hardship during the COVID-19 pandemic [3], and by the level of household food security.

Next, we conducted a reflexive thematic analysis [34,35] of 204 open-ended responses about respondents’ experiences with using SNAP. Informed by a literature review of existing qualitative research of SNAP participants’ experiences, the first author identified three constructs to use as codes for the analysis: barriers/facilitators to using SNAP, adequacy of SNAP benefits, and helpfulness of SNAP benefits. Two members of the study team met to discuss these codes and develop a coding strategy. Next, using Microsoft Excel, they independently assigned codes to the survey responses that described a given construct. The same team members met to discuss discrepancies, revised their approach when needed, and recoded the data until agreement was reached. Next, the two team members, for each period of data collection, generated themes independently, met to refine and finalize themes, and selected representative quotes to include in the study results.

## 3. Results

**Figure 3:**
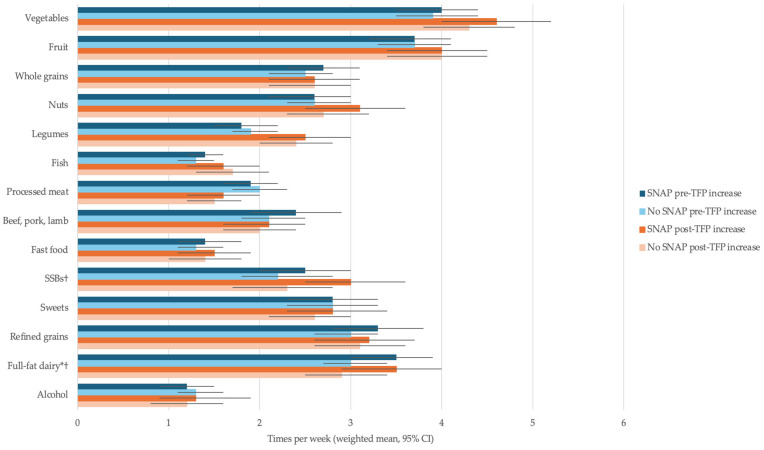
Adjusted mean food frequencies by SNAP participation before (October 2020–January 2021) and after (December 2021–February 2022) the Thrifty Food Plan increase among 3047 Massachusetts (U.S.) adults with low incomes. Pre-Thrifty Food Plan increase: SNAP (*n* = 457), No SNAP (*n* = 1229). Post-Thrifty Food Plan increase: SNAP (*n* = 527), No SNAP (*n* = 834). Estimates are adjusted for all other covariates (individual-level covariates of age, gender, educational attainment, race/ethnicity, and current employment status; and household-level covariates of food security status in the last 30 days, use of food pantry in the last 30 days, use of the Special Supplemental Nutrition Program for Women, Infants, and Children in the last 30 days, region, household size, presence of children in household, and income as a percentage of the federal poverty level). * *p* < 0.05 for differences in average intake by SNAP participation before the Thrifty Food Plan increase. † *p* < 0.05 for differences in average intake by SNAP participation after the Thrifty Food Plan increase. SSBs = Sugar-sweetened beverages. SNAP = Supplemental Nutrition Assistance Program. TFP = Thrifty Food Plan.

Demographic information for the analytical sample can be found in Table 1. A third of respondents reported participating in SNAP before the Thrifty Food Plan benefit increases compared to about half after the increase. Most respondents were adults less than 55 years of age, identified as non-Hispanic White, and had less than a college degree. More than half of respondents were female and about half were currently employed. Before the Thrifty Food Plan increase, SNAP participants differed from non-participants by employment status (*p* < 0.001) and use of food pantries (*p* < 0.0001). After the Thrifty Food Plan increase, SNAP participants differed from non-participants by the same covariates and also by gender (*p* < 0.01), educational attainment (*p* < 0.05), use of the Special Supplemental Nutrition Program for Women, Infants, and Children (*p* < 0.0001), and income as % of the federal poverty level (*p* < 0.05). Adjusted food frequencies by SNAP participation are displayed in Figure 3. Intake of different foods and beverages was largely similar between SNAP and eligible non-SNAP respondents, except for full-fat dairy, which was higher among SNAP participants before the revised Thrifty Food Plan (*p* < 0.05), and sugar-sweetened beverages, which was higher among SNAP participants after the benefit increases (*p* < 0.001).

There was no significant difference among SNAP participants in overall PDQS, PDQS sub-scores, or household food security status from before to after the 21% increase to SNAP benefits, compared to differences among SNAP-eligible non-participants, based on the difference-in-differences estimator (Table 2). Similarly, null results were found in the models of food frequencies and the sensitivity analyses for differences by the presence of children in the household and level of food security (data available by request).

We generated eight themes related to experiences with using SNAP across the coded survey responses from before and after the increase to SNAP benefits. Themes are displayed in Table 3 with illustrative quotes.

Four themes focused specifically on increases to SNAP benefits. Respondents expressed gratitude for the additional SNAP benefits, both from the 21% increase following the Thrifty Food Plan policy change and pandemic emergency allotments (theme 1). Participants commented, “I appreciate the extra amount they sent during the pandemic especially now that the children are remote learning we go through more food,” and “The extra COVID SNAP is very helpful.” Some respondents reported that the extra SNAP benefits allowed for food purchases that better met their dietary needs and preferences, whereas others still struggled to obtain the foods they wanted (theme 2). Example quotes after the Thrifty Food Plan increase included “I am SO VERY GRATEFUL for my benefits, since it means I can afford to buy higher quality food on a much more frequent basis because of the increase.” and “Forced to buy ‘junk’ type food like pasta and other wheat-based items to stretch the money.” Across both time points, respondents were anxious about losing the pandemic-era benefits or SNAP in general (theme 3): “I’m extremely worried about affording food when the pandemic benefits are no longer available.” Similarly, respondents worried about the rising costs of food (theme 4): “Even though SNAP increased, so did the cost of everything.”

Four themes described experiences with using SNAP in general both before and after the 21% increase to benefits. Many respondents noted being unable to meet food needs despite receiving SNAP (theme 5): “As a family of four, we do not have enough assistance to buy healthy foods,” and “My monthly SNAP does not give me enough for a family of 4.” However, participants consistently remarked that SNAP has been essential to financial survival (theme 6): “SNAP is a godsend. It allows me to get my bills paid on time.” Respondents also approved of the recent addition of SNAP online delivery (theme 7): “Online ordering has helped because I don’t drive.” However, some voiced technical challenges such as “More stores need to offer online buying with SNAP.” Another area for improvement cited by respondents included the high administrative burden of SNAP and fluctuations in benefit eligibility (theme 8). One respondent commented, “Wish DTA [Department of Transitional Assistance] caseworkers were easier to contact,” and another said, “My benefits were stopped so I had to reapply.”

We identified several consistencies in the status of adult diet quality and household food insecurity before and after the 21% permanent increase to SNAP benefits across the qualitative and quantitative findings. First, the qualitative findings described in theme 1 (i.e., increased benefits were still not enough to consistently meet household food needs) were also reflected in the results of the difference-in-differences analysis—there were no significant differences in household food security by SNAP participation before the Thrifty Food Plan increase or from before to after the increase. In theme 2, opinions were mixed among SNAP participants as to whether the increased benefits allowed them to purchase nutritious foods. These findings are consistent with the quantitative findings that adult diet quality did not differ by SNAP participation before the Thrifty Food Plan increase or from before to after the increase. In theme 4, the SNAP participants described how increased SNAP benefits were not enough to offset rising food costs and inflation, and they, thus, still experienced food insecurity. Similarly, additional gains in household food security were not observed among SNAP households compared to SNAP-eligible households in the difference-in-differences analysis.

## 4. Discussion

Despite the historic increase to SNAP in October 2021, following the Thrifty Food Plan reevaluation, our study found that this 21% increase in benefits—equivalent, on average, to a $36 increase in monthly benefits [18]—did not improve average diet quality or increase the probability of experiencing household food security for SNAP participants in Massachusetts during the months following the policy change. While respondents emphasized how SNAP benefits were essential to their household’s ability to access food, others described how the benefit amounts were still not enough to mitigate food insecurity. Additionally, while some were able to purchase more nutritious foods following the benefit increase, others described needing to consume foods of lower nutritional quality to stretch their SNAP benefits.

Respondents frequently cited rising food costs and inflation as major reasons for persistent food insecurity. Food prices increased by 3.9% in 2021 with the greatest increases beginning in the summer through the end of the calendar year [36], and inflation rates spiked by 7.5% for consumer items (7% for foods alone) which marked the largest annual increase in decades [37]. Adults with low incomes also reported disruptions in diet during this time due to the COVID-19 pandemic [27]. These economic conditions track with our qualitative data, where participants commented on how external factors prevented the additional SNAP benefits from stretching as far each month. These findings are also consistent with the only national study of the Thrifty Food Plan reevaluation and diet which found no significant impacts on diet quality or food security among adults with low incomes; however, the study’s qualitative data also indicated the helpfulness of the SNAP benefits during a time of rising inflation [20]. Similarly, a separate qualitative study of SNAP participants conducted in the summer of 2022 reported how pandemic-era SNAP benefits were still not enough to offset inflation [38]. It is possible the SNAP increases could have prompted widespread improvements in food security and diet quality in an alternate economic environment. However, particularly when considered in the international context, where we see several other Organisation for Economic Co-operation and Development (OECD) countries with more generous social safety net benefits to address poverty [8], it may also be that the increase simply was not large enough to meaningfully change food security and diet quality outcomes, regardless of the unusual economic changes that were co-occurring. Future research to quantify how food prices and inflation impact diet quality, as well as whether large amounts of cash or cash-like benefits produce better outcomes more generally, could yield useful insight into how further adjustments to the Thrifty Food Plan might advance population health.

Notably, while some respondents indicated being able to purchase more nutritious foods following increases to SNAP, others described needing to make tradeoffs between the costs and the nutritional value of foods. Though the quantitative analysis found higher intake of sugar-sweetened beverages among SNAP participants compared to non-participants at both time points, increases to SNAP benefits did not worsen diet quality for SNAP participants—a common argument against such increases is that participants will spend additional funds on less nutritious foods [39]. Further investigation of the adequacy of the SNAP benefit is warranted to determine what amount is needed or how SNAP could be restructured to ensure participating families have adequate access to food and to support better diet quality. Possible policy solutions could be to increase SNAP benefit amounts [19], expand nutrition incentive programs [19], and prevent targeted marketing of sugar-sweetened beverages which disproportionately impacts SNAP participants [40].

Importantly, this study’s qualitative findings offer insight into the experiences of SNAP participants, beyond the Thrifty Food Plan reevaluation, that hold implications for policy and practice. Some respondents indicated the option to use SNAP online for grocery delivery minimized barriers to shopping, though many voiced challenges to uptake, consistent with prior evidence [41,42], such as inadequate access to participating retailers, fees for delivery and tipping, and dissatisfaction with available foods. Participants in our study suggested a potential solution could be to require more food retailers to accept online electronic benefit transfer. Respondents also struggled with the administrative burden of using SNAP which can place individuals at risk of frequently leaving and then re-enrolling in the program. Policy changes to streamline caseloads and eligibility criteria could help to ensure uninterrupted participation [43]. These findings, combined with data from similar studies of SNAP participants, could help policymakers and practitioners refine program operations to increase participation in SNAP and improve the experience of using benefits.

This study is the first to explore experiences with using SNAP before and after the Thrifty Food Plan reevaluation in Massachusetts, a state with high levels of food insecurity [23], but it has several limitations. First, the authors did not have access to data from repeated measures of the same individuals; however, many households cycle on and off of SNAP [43] which could have presented challenges with data collection over a 12-month period. Second, self-reported participation in nutrition assistance programs is often under-reported [44], and thus, respondents may have been misclassified; the use of briefer assessments of both food insecurity (via the 6-item USDA Food Insecurity module) and diet quality (via the PDQS), while reducing respondent burden, may have also resulted in some nondifferential misclassification due to the fact that these measures, while valid, may have less precision. Results also may not be generalizable to SNAP participants outside of Massachusetts and are limited to the U.S. context. Third, data collection occurred during the COVID-19 pandemic when households saw a surge in charitable food initiatives [45] and food assistance efforts [32] which could have influenced the household food environments of participants. Fourth, although the regression models controlled for a range of potential sociodemographic confounders, there may have been other unmeasured differences between SNAP participants and SNAP-eligible non-participants that could have biased the results of the quantitative analyses. For example, compared to SNAP participants, eligible non-participants may have fewer hardships, face greater administrative burden to participation in SNAP, or have less knowledge and awareness of SNAP [46]. Last, missing data in our sample, if determined to be systematic, could have prevented our sample from being representative of Massachusetts adults with low incomes.

## 5. Conclusions

SNAP participants received a critical 21% percent increase to their monthly benefits during a period of economic turmoil resulting from inflation rates and the COVID-19 pandemic. While the increased benefits did not significantly improve diet quality or food insecurity among this study’s sample of Massachusetts adults with low incomes, write-in survey comments from SNAP participants underscored the integral role of SNAP in helping families acquire food despite their benefits not stretching as far. Further research is needed to generate evidence of how inflation and food prices impact nutrition outcomes so that policymakers and practitioners can ensure SNAP benefit amounts promote household food security and population health.

## Figures and Tables

**Table 1:** Survey-weighted characteristics of repeated cross-sectional samples of 1852 adults with low incomes in Massachusetts (U.S.) (2020–2022).

Characteristics	Pre-Thrifty Food Plan Increase ^1^ (*n* = 1051)			Post-Thrifty Food Plan Increase ^2^ (*n* = 801)		
	SNAP (*n* = 361)	No SNAP (*n* = 690)	*p*-Value	SNAP (*n* = 415)	No SNAP (*n* = 386)	*p*-Value
Adult demographics, % (95% CI)						
Age, in years			0.06			0.21
18–34	38.9 (30.8, 47.0)	46.3 (40.2, 52.4)		42.8 (35.4, 50.1)	49.9 (41.9, 58.0)	
35–54	39.0 (31.2, 46.9)	26.2 (21.0, 31.4)		38.2 (31.5, 44.8)	27.3 (20.7, 33.9)	
55–64	12.9 (8.3, 17.5)	12.8 (8.6, 17.1)		10.3 (6.3, 14.4)	11.8 (6.7, 16.9)	
65+	9.2 (3.1, 15.2)	14.6 (9.4, 19.9)		8.8 (4.4, 13.1)	11.0 (5.7, 16.2)	
Gender			0.26			<0.01
Female	53.4 (45.1, 61.7)	59.5 (53.3, 65.7)		65.9 (63.1, 76.0)	53.5 (45.3, 61.6)	
Educational attainment			0.63			<0.05
Some high school or less	31.5 (21.8, 41.1)	30.6 (23.3, 37.8)		32.7 (24.3, 41.1)	26.8 (16.9, 36.8)	
High school graduate or General Education Development	37.7 (30.4, 44.9)	36.2 (30.7, 41.6)		42.2 (35.5, 48.9)	37.3 (30.2, 44.4)	
Some college	17.2 (12.5, 21.9)	17.2 (13.8, 20.6)		16.0 (12.3, 19.7)	21.1 (16.3, 25.9)	
Associate’s degree	7.8 (4.9, 10.6)	6.9 (5.0, 8.8)		5.8 (4.2, 7.5)	5.3 (3.4, 7.1)	
Bachelor’s degree	3.8 (2.4, 5.3)	7.3 (5.5, 9.1)		2.4 (1.5, 3.4)	6.8 (4.8, 8.7)	
Graduate degree	2.1 (0.9, 3.2)	1.8 (1.0, 2.7)		0.9 (0.3, 1.4)	2.7 (1.2, 4.3)	
Race or ethnicity			0.83			0.11
Non-Hispanic White	57.7 (49.5, 65.9)	57.2 (51.0, 63.4)		61.4 (54.2, 68.7)	55.1 (47.0, 63.2)	
Non-Hispanic Black	9.0 (5.3, 12.7)	11.2 (7.7, 14.7)		9.7 (5.8, 13.6)	16.2 (9.5, 22.8)	
Hispanic or Latino	23.6 (16.0, 31.2)	23.4 (17.6, 29.1)		21.4 (14.9, 27.8)	19.6 (12.6, 26.5)	
Non-Hispanic Asian	4.4 (0.6, 8.2)	4.9 (2.5, 7.2)		4.0 (1.4, 6.6)	7.7 (4.7, 10.7)	
Non-Hispanic Other	5.4 (2.0, 8.8)	3.3 (1.1, 5.6)		3.6 (0.0, 7.1)	1.4 (0.2, 2.7)	
Currently employed	32.1 (24.7, 39.5)	48.9 (42.7, 55.0)	<0.001	36.7 (30.1, 43.4)	48.1 (40.2, 56.1)	<0.05
Household demographics, % (95% CI)						
Size, mean ± SE	4.1 (0.5)	4.0 (0.2)	0.96	3.3 (0.1)	3.6 (0.3)	0.32
Households with children	47.7 (39.5, 55.9)	52.7 (46.6, 58.9)	0.33	48.9 (41.7, 56.1)	41.3 (33.5, 49.1)	0.16
Income as % of federal poverty level			0.18			<0.05
<100%	46.7 (38.4, 54.9)	39.9 (34.0, 45.7)		56.3 (49.0, 63.5)	42.8 (34.9, 50.8)	
100–200%	53.3 (45.1, 61.6)	60.1 (54.3, 66.0)		43.7 (36.5, 51.0)	57.2 (49.2, 65.1)	
Region in Massachusetts			0.20			0.11
Central	15.4 (9.7, 21.1)	10.3 (6.8, 13.7)		14.9 (9.4, 20.3)	10.8 (6.9, 14.7)	
Western	19.0 (11.8, 26.3)	16.3 (12.2, 20.5)		22.9 (16.5, 29.4)	16.9 (11.3, 22.5)	
Eastern	65.6 (57.5, 73.6)	73.4 (68.3, 78.5)		62.2 (54.9, 69.4)	72.3 (65.7, 78.8)	
Special Supplemental Nutrition Program for Women, Infants, and Children in last 30 days	9.6 (3.8, 15.4)	4.6 (1.7, 7.5)	0.09	12.5 (7.4, 17.6)	1.2 (0.3, 2.1)	<0.0001
Food pantry in last 30 days	28.7 (21.2, 36.2)	9.9 (6.8, 12.9)	<0.0001	23.4 (18.1, 28.8)	8.2 (4.1, 12.2)	<0.0001
Dietary outcomes						
Adult diet quality in last 30 days, mean ± SE						
Overall Prime Diet Quality Score	43.2 (0.4)	43.8 (0.3)	0.31	43.9 (0.3)	44.0 (0.4)	0.86
Healthy sub-score	14.3 (0.4)	13.9 (0.2)	0.28	14.3 (0.4)	14.2 (0.4)	0.80
Unhealthy sub-score	19.1 (0.4)	18.1 (0.3)	0.05	18.4 (0.4)	18.2 (0.4)	0.66
Household food insecure in last 30 days, % (95% CI)	65.8 (58.1, 73.5)	57.7 (51.5, 63.8)	0.11	62.4 (55.2, 69.6)	49.1 (41.1, 57.2)	<0.05

^1^ Data were collected between October 2020–January 2021. ^2^ Data were collected between December 2021–February 2022. SNAP = Supplemental Nutrition Assistance Program.

**Table 2:** Adjusted difference-in-differences models for diet quality and household food security by SNAP participation from before (October 2020–January 2021) to after (December 2021–February 2022) the Thrifty Food Plan increase among 1852 adults with low incomes in Massachusetts (U.S.).

Outcome in the Last 30 Days	SNAP Participation		Post-Thrifty Food Plan Increase		SNAP Participation × Post-Thrifty Food Plan Increase	
	Difference (95% CI)	*p*-Value	Difference (95% CI)	*p*-Value	Difference (95% CI)	*p*-Value
Overall PDQS	−0.5 (−1.5, 0.5) ^1^	0.36	0.4 (−0.6, 1.4) ^2^	0.45	0.3 (−1.1, 1.8) ^3^	0.65
Healthy sub-score	0.5 (−0.3, 1.3)	0.24	0.3 (−0.5, 1.2)	0.47	−0.2 (−1.5, 1.0)	0.71
Unhealthy sub-score	1.0 (0.0, 1.9)	<0.05	−0.1 (−0.9, 0.8)	0.86	−0.6 (−1.9, 0.8)	0.41
Household food insecurity	0.0 (−0.1, 0.1) ^4^	0.59	−0.1 (−0.2, −0.0) ^5^	<0.05	0.0 (−0.1, 0.2) ^6^	0.57

Pre-Thrifty Food Plan increase: SNAP (*n* = 361), No SNAP (*n* = 690). Post-Thrifty Food Plan increase: SNAP (*n* = 415), No SNAP (*n* = 386). Estimates are adjusted for all other covariates (individual-level covariates of age, gender, educational attainment, race or ethnicity, and current employment status; and household-level covariates of food security status in the last 30 days, use of food pantry in the last 30 days, use of the Special Supplemental Nutrition Assistance Program for Women, Infants, and Children in the last 30 days, region, household size, presence of children in household, and income as a percentage of the federal poverty level). ^1^ The adjusted difference in average PDQS between SNAP participants and non-participants before the Thrifty Food Plan increase. ^2^ The adjusted difference in average PDQS among SNAP non-participants from before to after the Thrifty Food Plan increase. ^3^ The additional difference in average PDQS among SNAP participants from before to after the Thrifty Food Plan increase, compared to the difference among non-participants. ^4^ The adjusted difference in the probability of experiencing household food insecurity for SNAP participants compared to non-participants before the Thrifty Food Plan increase. ^5^ The adjusted difference in the probability of experiencing household food insecurity for SNAP non-participants from before to after the Thrifty Food Plan increase. ^6^ The additional difference in the probability of experiencing household food insecurity for SNAP participants from before to after the Thrifty Food Plan increase, compared to the difference among non-participants. PDQS = Prime Diet Quality Score. SNAP = Supplemental Nutrition Assistance Program.

**Table 3:** Qualitative themes of experiences with using SNAP before and after the 21% permanent increase to SNAP benefits in October 2021 among adults with low incomes in Massachusetts (U.S.).

Qualitative Analysis Themes ^a^	Pre- or Post-Thrifty Food Plan Increase	Illustrative Quotes
Theme 1: Though participants expressed gratitude for recent increases to SNAP benefits, including SNAP emergency allotments and the Thrifty Food Plan increase, many noted the additional benefits were still not enough to consistently meet household food needs.	Pre-increase	“Extremely grateful for the extra pandemic assistance. My SNAP feeds me one meal a day for about 2 weeks, give or take. I have to ask friends and family for food assistance because SNAP just doesn’t give enough, EVER, to survive on.” “The extra benefits provided have been extremely helpful.”
	Post-increase	“The increase has been very helpful and hope it continues.” “[SNAP] should increase [its] benefits as long as we still have this pandemic going on right now.”
Theme 2: Participants reported mixed opinions of the degree to which increased SNAP benefits supported their household’s dietary needs in the context of nutrition or dietary preferences.	Pre-increase	“Before COVID I had to spend extra money out of pocket because I’m on a medical diet. With the current increase I can afford to buy most [of] everything I need.”
	Post-increase	“Increased benefits has helped. But the foods I want/bought previously are no longer available because of the pandemic & supply chain problem. Healthier wheat bread, fresh fruits, adult cereals, etc. aren’t available in stores. I eat too much white bread/rolls.” “I have celiac disease which makes certain foods more expensive…If they could increase the amount then I [could] buy my gluten free items.”
Theme 3: Participants worried about pandemic-era SNAP benefits being rescinded and about losing benefits in general.	Pre-increase	“My SNAP benefits were increased with the pandemic. They have been very helpful and welcomed. I worry about them being lessened.” “I am grateful for my benefits but I know I will be losing them next month.”
	Post-increase	“I am extremely grateful for the help but am literally terrified what will happen if it gets lowered.” “I’m extremely worried about affording food when the pandemic benefits are no longer available.”
Theme 4: Rising food prices and costs of living prevented most participants from accessing enough food despite increased benefits.	Pre-increase	“It’s helpful, but don’t receive enough, since pandemic started. Prices have risen.” “[It’s] very helpful. [Too] bad we couldn’t get a little bit more. Food is so pricey.”
	Post-increase	“I always run out towards the end of the month. Food has gone up.” “The cost of food has increased far beyond SNAP increases. The pervasiveness of food deserts in communities of color has been exacerbated.”
Theme 5: Participants generally described SNAP as a necessity to keep food on the table, but most still found the benefit amounts insufficient.	Pre-increase	“If I didn’t get the SMALL amount of SNAP benefits that I [get] we probably wouldn’t be able to eat much during the month.” “SNAP benefits are enough to feed a family for 1/2 of the month. But not the whole month.”
	Post-increase	“SNAP has been a godsend. My husband passed away in 2021 and my income dropped to a third of what I was receiving. Without SNAP I may not have had the money to feed my family.” “We could use more, perhaps 20% more. Everyone reading this shops, just look at prices.”
Theme 6: SNAP benefits were essential to financial survival and helped some participants manage other household expenses.	Pre-increase	“SNAP benefits are my lifeline right now.” “Even before COVID-19, I would not have been able to survive without SNAP.”
	Post-increase	“It is a real lifesaver of a program especially with the increase in SNAP benefits. It allows me to stay on top of my bills. It has been a necessity in being able to keep our heads financially above water.” “Using SNAP helps me buy food so l can pay other bills with the money I don’t use on buying food.”
Theme 7: Using SNAP online helped participants overcome barriers to retailer access and/or transportation, but some cited residual barriers (e.g., cost of tipping, delivery fees, foods offered).	Pre-increase	“I am thrilled that Amazon just started accepting SNAP. It means less time in the grocery store, as there is no delivery [for] fresh or frozen foods in my area from Amazon.” “A lot of places online only have junk food for SNAP. I wish more online stores offered healthy SNAP options.”
	Post-increase	“For most of the pandemic, there were almost no groceries that would allow for paying for food for pickup or delivery via SNAP. That has improved during the latter part of 2021 and current times.” “It is finally allowed for grocery delivery but I wish more places were added and [that] they allowed pickup (because tipping and paying for delivery is hard). Also I wish that online grocery stores were accepted. Or food plans.”
Theme 8: Inconsistent or unrealistic eligibility criteria and/or confusing or high administrative burdens made it difficult for some participants to acquire or keep SNAP benefits.	Pre-increase	“Easy to use but the qualifications I feel are not consistent.” “My partner has SNAP, but I do not. I don’t know how to apply for SNAP.”
	Post-increase	“I did not think I would qualify. My husband is disabled and on Medicaid. I am unemployed and it seems we are always told we ‘make too much money’ but yet we barely get by.” “My SNAP benefits just started about a week ago after being shut off during the pandemic due to me “being ABWOD” able body without any dependents. Then my high school student daughter at the time, was made ‘head of household’ and benefits were cut down to only her yet card was still in my name? Then when she didn’t do her recertification they were completely shut off. She was only in 11th grade at the time and it was really hard to find someone to help me so I just applied and was approved about a week or so ago.”

^a^ Qualitative data were analyzed for Massachusetts adults who reported participating in SNAP in the last 30 days (*n* = 122 at pre-Thrifty Food Plan increase, *n* = 82 at post-Thrifty Food Plan increase). Supplemental Nutrition Assistance Program = SNAP.

## Data Availability

Data described in the manuscript cannot be made available because The Greater Boston Food Bank Research Division must approve all data requests at https://www.gbfb.org/contact-us/ (accessed on 26 April 2026).

## Abbreviations

The following abbreviations are used in this manuscript:

PDQS	Prime Diet Quality Score
SNAP	Supplemental Nutrition Assistance Program
SSBs	Sugar-Sweetened Beverages
TFP	Thrifty Food Plan
U.S.	United States
